# Utilizing the Most Accurate Preoperative Risk Calculator

**DOI:** 10.7759/cureus.17054

**Published:** 2021-08-10

**Authors:** Wasey Ali Yadullahi Mir, Francesco Fiumara, Dhan B Shrestha, Suman Gaire, Larissa Verda

**Affiliations:** 1 Department of Internal Medicine, Mount Sinai Hospital, Chicago, USA; 2 Department of Internal Medicine, University of Miami Palm Beach, Atlantis, USA; 3 Department of Emergency Medicine, Palpa Hospital, Palpa, NPL

**Keywords:** quality improvement, venous thromboembolism, myocardial infarction, postoperative complications, surgeons

## Abstract

The most commonly used preoperative assessment tools include the American College of Surgeons National Surgical Quality Improvement Program and the Revised Cardiac Risk Index. These tools seek to predict the risk of an individual experiencing postoperative complications, including but not limited to mortality, myocardial infarction, pneumonia, stroke, venous thromboembolism, and pneumonia. Many published studies have sought to objectively quantify the utility of the preoperative risk calculations by retrospectively compiling data for patients who underwent the same or comparable surgeries to compare actual complications to predicted complications. Therefore, we searched these studies to review the literature to draw more general conclusions and recommend which risk calculator is best for different types of surgeries.

## Introduction and background

Patients undergoing major noncardiac surgery are also at an increased risk of perioperative cardiovascular events. The risk depends both on the nature of the noncardiac surgery and the patient’s health status. In a study that included more than 10 million hospitalizations for major noncardiac surgery, the incidence of a major adverse cardiovascular and cerebrovascular event was found to be 3% [1]. Therefore, all patients undergoing noncardiac surgery should be assessed for the risk of perioperative cardiovascular events and measures should be taken for prevention, if necessary. There are multiple tools available for the assessment. Two of the most commonly used tools are the Revised Cardiac Risk Index (RCRI) and the American College of Surgeons National Surgical Quality Improvement Program (ACS-NSQIP).

RCRI consists of the following six independent predictors of major cardiac complications: a high-risk surgery, history of ischemic heart disease, history of heart failure, cerebrovascular disease, diabetes mellitus requiring insulin, and preoperative serum creatinine >2.0 mg/dL. In addition, the risk of cardiac complications is estimated based on the number of risk factors present [2]. RCRI was initially validated in a cohort of 1,422 individuals and has been subsequently evaluated by other studies [2,3].

The ACS-NSQIP consists of the following five perioperative myocardial infarction or cardiac arrest predictors: the type of surgery, dependent functional status, abnormal serum creatinine, age, and the American Society of Anesthesiologists class [4].

In this study, we reviewed 16 case series in detail that assessed the utility of these risk assessments by querying the PubMed database. The validity of the conclusions was evaluated by analyzing the results as a whole. Both indices were reviewed for intra-abdominal, vascular, transplant, neurosurgeries, and noncardiac surgeries. We sought to examine the conclusions of each statistical measure to determine whether the outcome for that risk calculator was predictable, not predictable, or unpredictable.

## Review

National Surgical Quality Improvement Program

Intra-abdominal Surgery

The NSQIP was not reliable for predicting outcomes. For open abdominal aortic aneurysm repair and endovascular aneurysm repair, predictability was 0.51 and 0.02, respectively. The predictability for open or robotic radical cystectomy using the NSQIP calculator was neither effective nor better than existing models [5].

Vascular Surgery

The NSQIP was ineffective for predicting the postoperative outcomes of vascular surgeries. In addition, the NSQIP was unreliable for predicting outcomes in both carotid endarterectomies and infra-inguinal lower extremity bypasses, showing predictability of 0.45 and 0.054, respectively [6].

Transplant Surgery

Canbolat et al. utilized the NSQIP in patients undergoing living donor liver transplantation and concluded no correlation between the NSQIP and the postoperative outcome of adverse events [7].

Neurosurgery

Although not perfect, the NSQIP is a better risk calculator for neurosurgeries. It was described as not predictive in geriatric patients undergoing lumbar surgery, with a Brier score of 0.321. Notwithstanding, the NSQIP proved to be accurate for predicting adverse outcomes in patients undergoing elective craniotomies for the small sample size in the study by Rutowski et al. [8]. Despite the low power of the former study, the NSQIP was described by Veeravagu et al. to be effective for predicting adverse outcomes in patients undergoing spinal surgery, highlighting an area under the curve (AUC) of 0.669 in larger sample size [9].

Biomarkers in Noncardiac Surgery

One study utilized the NSQIP to assess patients undergoing noncardiac surgery. Markovic et al. used the area under the receiver operating characteristic curve to analyze whether the NSQIP could accurately predict outcomes when compared with the biomarkers baculoviral IAP repeat-containing protein 5, heart-type fatty acid-binding protein, and high sensitivity C-reactive protein. The results showed an AUC of 0.941 when combining the NSQIP with the three biomarkers, indicating excellent predictability [10].

Revised Cardiac Risk Index

Intra-abdominal Surgery

The RCRI demonstrated mixed results for predicting outcomes. It did not reliably predict postoperative outcomes in either open abdominal aortic aneurysm repairs or endovascular aneurysm repairs [6]. However, it was reliable for predicting adverse outcomes in the studies describing major digestive, breast, or vascular surgery in patients >65 years old [11] and endovascular abdominal aortic aneurysm repair [12].

Vascular Surgery

The RCRI overall proved to be ineffective for predicting outcomes. It was unreliable when predicting outcomes in both carotid endarterectomies and infra-inguinal lower extremity bypasses, showing predictability of 0.17 and 0.002, respectively. Although the statistical measure showed some positive association using the AUC, in three of the five studies, the RCRI was determined to be unreliable. RCRI demonstrated predictability in noncardiac vascular surgery (odds ratio [OR] = 3.9) and vascular surgery (OR = 1.73) [13,14]. Both of these studies used OR as the statistical measure of choice. It should be considered that if the three studies describing AUC results of <0.70 and >0.55 utilized OR as well, there may have been significant findings.

Transplant Surgery

According to a study, the RCRI was not predictable, describing a weakly positive correlation of 0.245. Conversely, it was reliable and successful in predicting adverse outcomes in both liver and kidney transplants. Park et al. measured adjusted OR to be 4.35, indicating a positive result for RCRI patients who underwent liver transplantation [15]. Hoftman et al. described an AUC of 0.77 for patients undergoing kidney transplantation [16].

Neurosurgery

The RCRI was found to be predictive for patients undergoing elective craniotomies, as described by Rutkowski et al., utilizing a small sample study [8]. However, Carabini et al., utilizing a significantly larger study sample, reported the RCRI to be unreliable for predicting adverse outcomes in patients undergoing spinal fusion with instrumentation, reporting an AUC of 0.54 [17].

Biomarkers in Noncardiac Surgery

RCRI was found to have resoundingly positive predictability when combined with specific biomarkers. The adjusted hazard ratio demonstrated predictability in the RCRI combined with N-terminal pro-B-type natriuretic peptide for noncardiac surgeries [18]. Kopec et al. utilized high-sensitivity cardiac troponin T (hs-cTnT) combined with the RCRI, resulting in an AUC of 0.716 for patients undergoing major noncardiac surgery. Interestingly, in the same study, when the RCRI was used on its own, without hs-cTnT, the AUC was found to be 0.59, a result that indicated unpredictability [19]. Lastly, Golubovic et al. utilized high-sensitivity troponin I in combination with the RCRI for patients undergoing major elective vascular surgery in which the AUC was 0.909 [20].

Based on the review of existing literature and objective analysis, the NSQIP fell short in reliably predicting postoperative complications for the majority of surgeries. Conversely, the RCRI was more reliable in accurately predicting postoperative complications. The best preoperative risk calculator recommendations for each type of surgery are outlined in Table 1.

**Table 1:** Category of surgery and the appropriate risk calculator. RCRI: Revised Cardiac Risk Index; NSQIP: National Surgical Quality Improvement Program

Category of surgery	Risk calculator
Intra-abdominal	RCRI
Vascular	RCRI
Transplant	RCRI
Neurosurgery	NSQIP
Noncardiac with biomarkers	Either or both

## Conclusions

The RCRI was determined to be the more effective risk calculator in predicting postoperative outcomes for intra-abdominal, vascular, and transplant surgeries. The NSQIP was determined to be the more effective risk calculator in predicting the postoperative outcomes for neurosurgical surgeries. In the case of noncardiac surgery, when supplemented with biomarkers, both the RCRI and NSQIP proved to be highly effective at predicting outcomes.
